# From Association to Academy: Embracing New Opportunities

**DOI:** 10.1093/geroni/igaa057.1811

**Published:** 2020-12-16

**Authors:** David Burdick

**Affiliations:** Stockton University, Port Republic, New Jersey, United States

## Abstract

This presentation will explore the transition from Association to Academy. This transition occurred as GSA governance was also changing, presenting significant challenges on several fronts. While some of these challenges will be considered, the presentation will primarily focus on AGHE’s many emerging opportunities and the steps various AGHE committees are taking to enhance the Academy’s role and relevance and fulfill its mission. The Advancement Committee’s project to assess educational programs across GSA sections, the Age-Friendly University initiative, Ageism First Aide, and other innovative programs moving forward will briefly be noted. Part of a symposium sponsored by the Geriatric Education Interest Group.

